# Benchmarking Q Fever Transmission in the Czech Republic and Serbia: A One Health Subnational Population Study

**DOI:** 10.1038/s41598-026-47183-5

**Published:** 2026-04-06

**Authors:** Ondřej Holý, Sara Savić, Jaroslav Bzdil, Jeadran Malagon, Ekaterina Ryzhova, Hana Dostálová, Michal Křupka, Tatjana Pustahija, Dejan Bugarski, Tereza Schovánková, Laith Hussain-Alkhateeb, Snežana Medić

**Affiliations:** 1 Science and Research Centre, Faculty of Health Sciences, Palacky University Olomouc, Hněvotínská 3, Olomouc, 775 15 Czech Republic; 2 Department of Health Care Sciences, Faculty of Humanities, Tomas Bata University in Zlin, Zlin, 76001 Czech Republic; 3 Scientific Veterinary Institute “Novi Sad”, Rumenački put 20, Novi Sad, 21000 Serbia; 4 Department of Biochemistry, Faculty of Science, Palacky University Olomouc, Šlechtitelů 27, Olomouc, Czech Republic; 5 Instituto Nacional de Salud, Avenida Calle 26 # 51–20, Bogotá, 11031 Colombia; 6 Department of Virology and Serology, State Veterinary Institute Olomouc, Jakoubka ze Stříbra 1, Olomouc, 779 00 Czech Republic; 7 Department of Immunology, Faculty of Medicine and Dentistry, Palacky University Olomouc, Hněvotínská 3, Olomouc, 775 15 Czech Republic; 8 Department of Epidemiology, Faculty of Medicine, University of Novi Sad, Hajduk Veljkova 3, Novi Sad, 21000 Serbia; 9 Center for Disease Control and Prevention, Institute of Public Health of Vojvodina, Futoška 121, Novi Sad, 21000 Serbia; 10 School of Public Health and Community Medicine, Institute of Medicine, Sahlgrenska Academy, University of Gothenburg, Gothenburg, Sweden; 11 Population Health Research Section, King Abdullah International Medical Research Centre, Riyadh, Saudi Arabia

**Keywords:** *Coxiella burnetii*, Animal, Human, Europe, Zoonosis, Farming practices, Vaccination, Diseases, Microbiology, Zoology

## Abstract

Q fever, caused by *Coxiella burnetii*, poses a zoonotic threat worldwide. Understanding its epidemiology in diverse settings is crucial for effective control measures. A retrospective observational study compared Q fever epidemiology in the regions of Moravia and Silesia (Czech Republic) and Srem and South Bačka districts of the Autonomous Province of Vojvodina (Serbia), from 2011 to 2018. Here, we analyzed the demographic and spatial patterns of human and animal Q fever retrieved from the human and veterinary official surveillance databases. Animal seroprevalence of *Coxiella burnetii* was broad but consistently high in Czech cattle (range 8.6–60.0%) and was highest in Žďár nad Sázavou, Brno-město, and Znojmo districts. Moravia and Silesia saw an increasing average Q fever seroprevalence in cattle (range 22.9–32.2%), while Srem and South Bačka districts of Vojvodina exhibited annual and seasonal fluctuations with varying seroprevalence in goats (0–14,7%), sheep (0–12,0%), and cattle (0–33,0%). Human Q fever cases were low in Moravia and Silesia (*n* = 3), in contrast to 76 cases and three outbreaks recorded in Srem district, accounting for approximately one-third of all cases and half of all outbreaks in Vojvodina that occurred in study years. The high seroprevalence of Q fever among cattle in Moravia and Silesia regions was not followed by human cases. In contrast, the endemic maintenance of Q fever among livestock in two districts of Vojvodina, was accompanied by an unfavourable situation in humans. A One Health approach including tailored interventions, such as vaccination of animals and safe farming practices, are essential for addressing Q fever effectively.

## Introduction

Q fever is an infection that affects livestock and farmer welfare around the world. Its causative agent, *Coxiella burnetii*, causes disease in both animals and humans^1^. Outbreaks of Q fever have been reported in different continents, including Europe^2–4^.Q fever in humans can be either acute or chronic^5^. Symptomatic cases typically present as a non-specific febrile illness^6^ that can progress to atypical pneumonia^5,6^. In humans, chronic infection can result in severe endocarditis and vascular infections^7^.The reservoirs of the disease encompass many wild and domestic mammals, especially cattle, sheep, and goats^7–9^, which serve as the most frequent reservoirs of infection for humans^10^. In most animals, Q fever is asymptomatic, but on some occasions, it had caused abortions^1,2^.

The epidemiological features of Q fever vary depending on the geographical area and level of endemicity^10^. The diversity of infection reservoirs, various transmission pathways hinder the development of unified approaches to prevent Q fever outbreaks^11^. The primary mode of acquiring *Coxiella burnetii* infection is through inhalation of a contaminated aerosol, and rarely with consumption of contaminated raw food such as milk. Various body secretions and excretions of infected animals, including milk and parturition products, contain *Coxiella burnetii*^12,13^. Wind patterns have been implicated in the spread of Q fever^14^. Research focused on factors contributing to endemic maintenance of Q fever in the Autonomous province of Vojvodina (Vojvodina), Serbia, highlighted a positive correlation between an increase in Q fever cases and stronger winds, which facilitate the dissemination of infected dust particles^15^. Although ticks may play a role in disease transmission^1,7^, in Serbia, this infection route is rare, due to a low prevalence of *Coxiella burnetii* in ticks^16^. In general, prevention measures for Q fever refer to vaccination of animals in high-risk areas and on-farm hygienic measures^17,18^.

This study is based on a comparative analysis and a critical assessment of the epidemiology of Q fever in humans and farm animals, including control measures, using surveillance data from Serbia (Vojvodina) and the Czech Republic (Moravia and Silesia regions). Reports from the Czech Republic indicate minimal human autochthonous Q fever cases^19^, while in Serbia, Q fever is an endemic disease predominantly registered in Vojvodina^15^ with frequent outbreaks^20,21^. The importance of including countries with an unequal burden of Q fever in the same analysis is in the identification of potential factors that may contribute to the endemic maintenance of the disease and outbreak occurrence, as well as the consideration of more effective measures that could help alleviate the burden of the disease in the most vulnerable areas.

## Materials and methods

Our study employed a retrospective, observational design utilizing population-level surveillance data, spanning from 2011 to 2018. The primary objective was to compare epidemiological parameters related to Q fever occurrence in both humans and animals across the study areas of two countries. Additionally, we conducted an inter-country evaluation of control strategies aimed at mitigating Q fever transmission.

### Study area and population

Northern Serbian province Vojvodina is situated in the Southeast of Europe and covers an area of 21,500 km² (corresponding to 24% of the total area of Serbia which covers 88,499 km^2^)^22^. Its population of 1.7 million inhabitants, constitutes almost a quarter of the population of Serbia^22^. Administratively, the province is divided into seven districts and surrounded by Croatia, Romania, Hungary, Bosnia and Herzegovina, and central Serbia. About 51% (≈ 890,000) of Vojvodina’s population lives in the Srem and South Bačka districts, which together comprise approximately 35% (7,511 km²) of the province’s land area^22^. These two districts are estimated to hold 40–50% of Vojvodina’s livestock population. Specifically, in Srem district, livestock data indicate approximately 33,000 cattle distributed across 3,054 households, as well as around 30,000 sheep and goats in 1,366 households. The One Health analysis was conducted in these two districts due to the availability of both human and animal data. The Czech study area includes separate administrative units of Moravia and Silesia in the eastern and northeastern parts of the Czech Republic, respectively. Moravia region extends over an area of ≈ 22,000 km² and is populated with ≈ 3 million citizens. Silesia region covers an area of ≈ 4,500 km² with a population of ≈ 1 million people^23^. Study areas are chosen because they differ in Q fever burden and control strategies but are comparable in several geo-climatic characteristics: landscape configuration, rich river networks, fertile soils and climate. In Vojvodina, the climate is continental, with cold winters, hot and humid summers, and even distribution of precipitation^15,24^. In autumn and winter, a strong, cold and dry southeast wind prevails in the Danube River basin of Vojvodina called “Košava” prevail^15^. The average annual temperature is 11–12 °C. Snow cover usually lasts from November to March. The humidity of the air reaches 70%^24^. Climate in Moravia and Silesia is mainly continental, with warm summers and cold, cloudy, and snowy winters. The average annual temperature reaches 10 °C. The average humidity in winter reaches 82%, in summer – 65%^23^.

### Sources of information and data

**Serbia.** Human Q fever: the notification of Q fever in Serbia is mandatory and case based. The classification of cases (probable or confirmed) is based on the European Center for Disease Prevention and Control (ECDC) Q fever case definition^25^. Only confirmed and probable Q fever cases were included in the study. Human morbidity data were obtained from the surveillance database of the Institute of Public Health of Vojvodina (IPHV). Data included variables such as patient demographics (gender and age), residence, date of symptom onset, clinical characteristics, hospitalization status, test of case confirmation, travel data, contact history, potential epidemiological links, and disease outcome. Annual reports from the official website of the Institute of Public Health of Serbia (IPHS) were also used^21^. Animal Q fever: data on Q fever cases in animals in Srem and South Bačka, obtained from the Serbian Veterinary Directorate of the Ministry of Agriculture, Forestry and Water Management, included the number and type of affected animals, registration years, and geographic locations of farms^26^. Only laboratory confirmed cases of *Coxiella burnetii* infection in animals were included. The seroprevalence was calculated as the ratio of seropositives in relation to the total number of tested animals (%). The control measures on farms in case of Q fever in animals were described.

**Czech Republic.** Human Q fever data were extracted from reports submitted to the ECDC^19^. Only laboratory confirmed human cases were included. We analyzed variables such as region of residence, gender, age, date of first symptoms, importation status including country of origin. Animal Q fever: veterinary data were collected by laboratories of state veterinary institutes and obtained with the approval of the State Veterinary Administration of the Czech Republic. Samples were collected within state-mandated monitoring. The data included the names of the farms, their districts, animal species, the number and type of samples taken, and the result of the laboratory testing. Testing of samples was conducted within the framework of monitoring zoonoses and zoonotic pathogens.

### Characteristics of cattle and small ruminant husbandry of the study areas

Small ruminant production in Serbia is predominantly characterized by small-scale farming systems, usually organized within family farms with relatively small herds. According to the national agricultural census, most farms keep fewer than 10 animals per species, and livestock production is mainly based on mixed or semi-extensive systems, often involving grazing and close contact between people and animals^27^. Although livestock production in Vojvodina is generally more market-oriented, small and medium-sized farms still dominate. While pig production is dominant in this region, sheep and goats are usually raised in semi-intensive or extensive conditions, often in peri-urban or pasture systems. Within the Srem district, which is characterized by a combination of arable land, pastures, and fragmented smallholdings, small ruminant farming is commonly practiced in close proximity to human settlements, often involving extensive grazing and seasonal lambing/kidding^28^.

Small ruminant production in Czech Republic:

Cattle production in the Czech Republic is highly specialized and technologically intensive, particularly in the dairy sector. Most dairy cows are kept in medium-sized to large herds in modern free-stall barns, with grazing usually limited to the vegetation season and indoor housing during winter. There are approximately 10,000–11,000 cattle holdings, with a total cattle population of about 1.3–1.4 million head, including 360,000–380,000 dairy cows^29,30^. Most animals are concentrated in large-scale farms. Beef cattle are reared mainly under pasture-based systems^31^. Small farms usually keep 10–50 head of cattle, medium-sized farms 50–200 head, and large enterprises 200–1,000 or more. The average herd size is around 120–140 head per holding, and 80–100 cows per dairy farm, representing one of the highest herd concentrations in the EU^31^. Large dairy farms predominantly use TMR (Total Mixed Ration), while smaller farms rely on hay, haylage, and concentrates. Water is supplied through pasture troughs and automatic drinkers in barns. Housing is mostly loose, with straw bedding or slatted floors with slurry channels; tie-stall housing is used on fewer than 15% of farms^31^. Average annual milk yield is approximately 9,500–10,500 kg per cow, ranging from about 6,000 kg on low-input farms to more than 12,000 kg on top-performing farms^31^. The main beef breeds are Charolais, Limousin, Hereford, and Aberdeen Angus, while the dominant dairy breeds are Holstein, Czech Fleckvieh/Simmental, and Jersey^31^.

Sheep and goat farming is of lower economic importance and is generally extensive, often focused on landscape management and seasonal grazing. Herds are usually small and frequently part of mixed farming systems. Most holdings keep fewer than 50 animals, and 80–90% of flocks are managed on pasture, with housing used mainly in winter or during lambing/kidding^30^. The Czech Republic has approximately 220,000–240,000 sheep in 6,000–7,000 holdings and 25,000–30,000 goats in 3,000–4,000 holdings^29,30^. Typical sheep flocks contain 10–30 animals, while professional farms may keep 200–800 head. Common sheep breeds include Merino, Suffolk, Texel, and Romanov, while goat production is dominated by White Shorthaired goats, followed by Brown Shorthaired and Anglo-Nubian breeds^30^. Feeding is mainly pasture-based, supplemented with hay in winter, while concentrates are typically used only in dairy animals. Housing, when provided, is usually loose with bedding^30^.

### Sample process and laboratory confirmation

Serum samples from patients displaying clinical symptoms of Q fever in Serbia were tested at the Serbian Reference Laboratory for Q fever, located at the IPH of Zrenjanin. Human cases of Q fever were confirmed using enzyme-linked immunosorbent assay (ELISA, NovaLisa)^32^. Serological confirmation relied on detecting IgM and/or IgG antibodies to *Coxiella burnetii* phase II antigen. Paired serum samples, taken at least two weeks apart, were examined if the initial serology results were equivocal or negative.

In Vojvodina, including Srem and South Bačka districts, animal sampling is carried out after reporting an abortion in cattle, sheep and goats. Moreover, sampling of asymptomatic cattle, sheep and goats is implemented in case of human Q fever outbreak investigation. Besides, animal samples underwent examination as part of the annual animal health monitoring in the Republic of Serbia. The investigation was conducted during the period 2012–2018, as no animal sampling or testing was performed in either district in 2011.The enzyme-linked immunosorbent assay (ELISA) was employed to detect antibodies against *Coxiella burnetii* in blood samples obtained from cattle and sheep, following the guidelines of the World Organization for Animal Health (OIE)^18^. Positive results were further confirmed using a Coxiella phase I or phase II confirmation kit (also ELISA). In the Czech Republic, monitoring of animal diseases is mandated by the Veterinary Act No. 166/1999 Coll., as amended. Sheep, goats, and cattle were included in the monitoring program, with blood samples collected within 10 days after abortion. Positive results underwent further examination using the complement fixation reaction (CFT) by accredited laboratories (ELISA- ID screen Q FeverIndirect).

### Control of Q fever in human and animal population

In Serbia, upon notification of human Q fever cases, control measures are immediately implemented in suspected herds, including the temporary prohibition of livestock trading, slaughter, and the use of unpasteurized milk and dairy products. These measures remain in place if any tested animals are positive or the herd is confirmed as infected and continue until negative serological and/or PCR results in tested animals are obtained. A herd is considered infected if at least one animal suspected of infection tests positive by PCR, or if two or more animals with abortions, reproductive disorders, or characteristic clinical signs yield positive serological results. In infected herds, additional measures include isolation of infected animals, identification and record-keeping of susceptible animals, enhanced biosecurity and hygiene, proper disposal of aborted fetuses and placenta, and restriction of milk and dairy product use unless pasteurized. These measures continue until infection is ruled out in the subsequent reproductive cycle, or for at least six months from confirmation of Q fever^33^. PCR testing of animal samples is rarely used, after obtaining inconclusive serology, in case of livestock abortions and/or during the investigation of reported human Q fever outbreaks. Human blood donors from affected regions are excluded, with hospitals implementing precautionary measures. Health education campaigns for farmers emphasize appropriate hygiene practices when handling livestock by-products and safe procedures for clothing and footwear. In the Czech Republic, systematic control of Q fever in the animal population has not yet been implemented. The disease is still considered unusual, rare, and relatively unknown, particularly in human medicine in Czech Republic.

### Data analysis

Data were analyzed demographically, chronologically, and topographically for the observed period (2011–2018). This included analyzing the annual number of reported human Q fever cases, age and gender distribution of cases, crude annual incidence rates (per 100,000 people), and disease trends. Additionally, the number and characterization of Q fever outbreaks in the human population were analyzed at the national level (Serbia), and at the provincial level (Vojvodina) for descriptive purposes. We further examined the proportion (%) of cases registered in outbreaks and the presumptive route of transmission. In line with the One Health approach, the integrated analysis of human and animal data was restricted to the two districts (Srem and South Bačka), where both datasets were available. Statistical data processing involved descriptive analysis of variables: age (age groups: ≤ 19, 20–29, 30–39, 40–49, 50–59 and > 60 years), gender, residence of human Q fever cases, annual distribution of human cases and outbreaks, month of case notification, and seropositive animals (n, %) by type and locations of herds in the study region. Statistical analyses were conducted using SPSS software, version 21.0. Mapping of data was performed using Quantum GIS (QGIS) software, version 3.4, to visualize geographical distribution.

## Results

### Q fever in animal population

The seroprevalence of *Coxiella burnetii* among cattle in the Moravia and Silesia regions of the Czech Republic is presented in Table 1. While cattle represent a substantial population, goats and sheep, are significantly smaller in number and tested seronegative during the study period. A total of 8454 blood samples were obtained, of which 8362 were from cows, 49 from goats, and 43 from sheep.

**Table 1:** Seroprevalence of (%) of *Coxiella burnetii* in cattle, 2011–2018 in districts of Moravia and Silesia regions, Czech Republic.

District	2011		2012		2013		2014		2015		2016		2017		2018		District Average
	n	%	n	%	n	%	n	%	n	%	n	%	n	%	n	%	%
Blansko	9	*22.5*	15	*42.9*	17	*42.5*	5	27.8	4	*33.3*	12	38.7	10	*26.3*	*5*	23.8	*32.2*
Brno-město	NT		NT		0	0.0	1	100	NT		0	0.0	1	*100*	*NT*		*50.0*
Brno-venkov	14	*31.1*	8	*20.5*	12	*26.7*	*15*	*34.9*	15	*33.3*	*16*	*34.8*	10	*30.3*	10	*28.6*	*30.0*
Břeclav	15	*28.8*	13	*26.5*	13	*31.0*	*11*	*29.7*	21	*36.8*	*11*	*29.7*	12	*25.0*	9	*30.0*	*29.7*
Hodonín	6	*17.6*	3	*15.0*	6	*35.3*	*4*	*33.3*	4	*30.8*	*3*	*33.3*	3	*27.3*	1	*14.3*	*25.9*
Vyškov	5	*21.7*	1	*20.0*	2	*33.3*	*0*	*0.0*	1	*14.3*	*2*	*66.7*	1	*16.7*	4	*44.4*	*27.1*
Znojmo	NT		NT		NT		0	0.0	1	100	1	33.3	4	*57.1*	*2*	*28.6*	*43.8*
Třebíč	NT		NT		NT		NT		NT		0	0.0	NT		1	*20.0*	*10.0*
Žďár nad Sázavou	0	*0.0*	*NT*		NT		1	100	NT		NT		2	*100*	*2*	*40.0*	*60.0*
Bruntál	6	*14.3*	3	*8.8*	5	*13.9*	*3*	10.7	5	*14.3*	5	*16.1*	*0*	*0.0*	4	*18.2*	*12.0*
Frýdek Místek	27	*37.5*	19	*23.8*	20	*26.3*	*18*	*32.7*	14	*17.9*	18	*26.5*	*23*	*34.3*	17	*29.3*	*28.5*
Karviná	9	*26.5*	7	*20.0*	11	*29.7*	*6*	*22.2*	7	*17.9*	6	*20.0*	7	*35.0*	3	*10.7*	*22.8*
Nový Jičín	26	*24.1*	23	*25.6*	28	*23.5*	*33*	*30.6*	23	*21.3*	22	*22.7*	22	*25.9*	25	*28.4*	*25.3*
Opava	16	*27.1*	10	*17.2*	17	*26.6*	*10*	*19.6*	11	*22.4*	9	*23.1*	10	*27.0*	16	*33.3*	*24.5*
Ostrava-Město	2	*25.0*	5	*33.3*	6	*31.6*	*7*	*46.7*	5	*45.5*	5	*55.6*	1	*11.1*	2	*33.3*	*35.3*
Jeseník	1	*12.5*	0	*0.0*	1	*14.3*	*1*	*16.7*	0	*0.0*	0	*0.0*	0	*0.0*	1	*25.0*	*8.6*
Olomouc	31	*24.2*	19	*18.4*	24	*18.9*	*30*	*18.6*	26	*19.3*	25	*22.5*	33	*25.8*	32	*29.6*	*22.2*
Prostějov	18	*27.3*	10	*31.3*	12	*21.8*	*17*	*20.5*	11	*19.3*	17	*35.4*	13	*31.7*	11	*31.4*	*27.3*
Přerov	24	*37.5*	20	*44.4*	22	*30.6*	*23*	*31.1*	23	*37.7*	13	*32.5*	13	*23.2*	13	*33.3*	*33.8*
Šumperk	18	*24.3*	14	*24.6*	18	*24.7*	*25*	*25.3*	19	*39.6*	25	*35.7*	15	*28.8*	16	*36.4*	*29.9*
Kroměříž	39	*26.4*	14	*16.1*	27	*35.1*	*18*	*22.2*	17	*24.6*	26	*28.3*	27	*26.5*	31	*24.6*	*25.5*
Uherské Hradiště	7	*18.4*	13	*29.5*	18	*41.9*	*4*	*19.0*	6	*14.0*	7	*30.4*	12	*42.9*	12	*27.3*	*27.9*
Vsetín	14	*31.6*	10	*18.2*	11	*19.6*	*17*	*28.3*	15	*30.0*	11	*22.4*	10	*19.2*	9	*23.7*	*24.1*
Zlín	8	*16.7*	9	*21.4*	11	*35.5*	*14*	*48.3*	12	*33.3*	3	*15.0*	8	*27.6*	11	*26.8*	*28.1*
Year Average	14	*23.6*	11	*22.9*	13	*26.8*	*11*	*31.2*	11	28.8	10	*28.8*	10	*32.2*	10	*27.9*	-
Year Total	295		216		281		263		240		237		237		237		-

n – number of positive cases, NT - not tested.

**Fig. 1:**
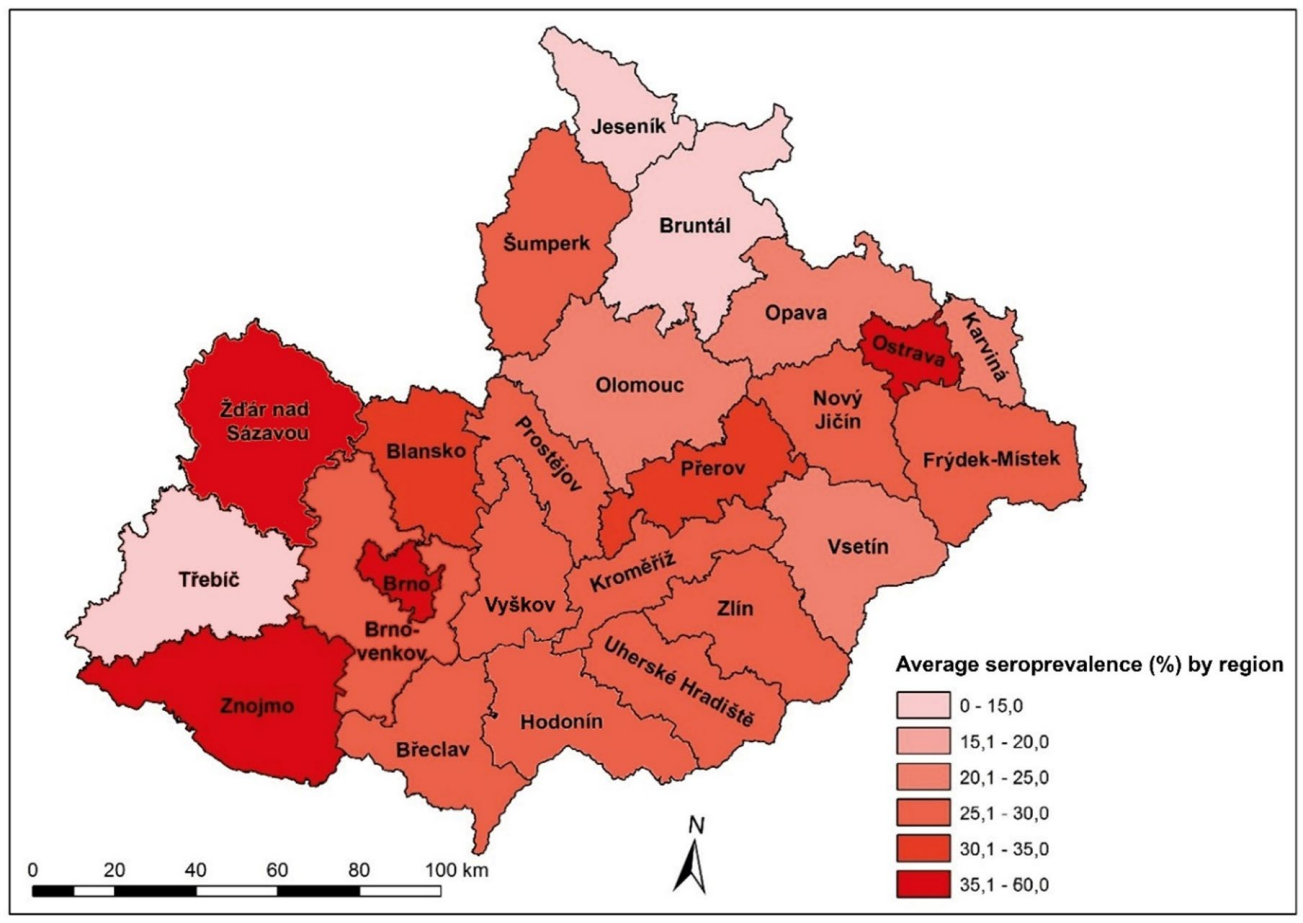
The average seroprevalence of *Coxiella burnetii* among cattle during the 2011–2018 period by districts of Moravia and Silesia regions, Czech Republic.

In 2018, the total cattle population amounted to 1,415,770, whereas sheep and goats numbered only 218,915 and 30,316, respectively. An increase in cattle numbers by 1,462 heads was specifically established within the Czech study areas from 2016 to 2018, while the total number of cattle remained stable at the country level. Although, no areas with zero seroprevalence of the Q fever pathogen in cattle were identified, the seroprevalence was highest in southwestern Moravia, particularly in the regions of Žďár nad Sázavou, Brno-město, and Znojmo (Fig. 1).

Also, the average annual percentage of seropositive cattle out of all examined cattle in Moravia and Silesia increased linearly (from 22,9% in 2012 to 32,2% in 2017) in the observed period.

Between 2012 and 2018, total of 6,134 animals were tested across Srem and South Bačka of which 9.8% were seropositive (Table 2). Seropositivity was highest in cattle, reaching 32.2% in South Bačka (2014) and 33.3% in Srem (2012). In South Bačka, no seropositive goats were detected, and sheep were positive only between 2016 and 2018 (overall 7.0%).

In Srem, serorevalence varied among goats (range 0-14.7%), sheep (0–12.0%), and cattle (0–33.0%) over the years. Seropositive goats were first detected in 2017 and 2018 associated with a Q fever outbreak on the goat farm in the village Kukujevci in Srem district. Sheep showed fluctuations in seroprevalence, reaching the highest level (12.0%) in 2017, while cattle displayed an overall seroprevalence of 15.8%, with peaks recorded in 2012–2014 (Table 2).

**Table 2:** Seroprevalence of *Coxiella burnetii* (%) among goats, sheep, and cattle tested in the period 2012–2018 in South Bačka and Srem districts, Vojvodina, Serbia.

Animal	District^1^	2012			2013			2014			2015			2016			2017			2018			Total		
		T^2^	*P* ^3^	%^4^	T	*P*	%	T	*P*	%	T	*P*	%	T	*P*	%	T	*P*	%	T	*P*	%	T	*P*	%
Goat	South Bačka	0	0	0.0	0	0	0.0	0	0	0.0	13	0	0.0	15	0	0.0	30	0	0.0	27	0	0.0	85	0	0.0
	Srem	0	0	0.0	0	0	0.0	1	0	0.0	157	0	0.0	24	0	0.0	68	10	14.7	120	14	11.7	370	24	6.5
Sheep	South Bačka	0	0	0.0	48	0	0.0	72	0	0.0	83	0	0.0	587	35	6.0	836	72	8.6	1069	81	7.6	2695	188	7.0
	Srem	0	0	0.0	25	2	8.0	96	0	0.0	81	4	4.9	187	3	1.6	490	59	12.0	371	11	3.0	1250	79	6.3
Cattle	South Bačka	260	19	7.3	206	24	11.7	124	40	32.2	240	53	22.1	544	137	25.2	278	29	10.4	25	1	4.0	1677	303	18.1
	Srem	9	3	33.3	7	2	28.6	4	1	25.0	13	1	0.0	9	0	0.0	13	2	15.4	2	0	0.0	57	9	15.8
Total		269	22	8.2	286	28	9.8	297	41	13.8	587	58	9.9	1366	175	12.8	1715	172	10.0	1614	107	6.6	6134	603	9.8

^1^No data for the rest of the districts in Vojvodina province;

^2^Total number of animals sampled;

^3^ Seropositive for *Coxiella burnetii*.

^**4**^% Ratio of seropositives.

**Fig. 2:**
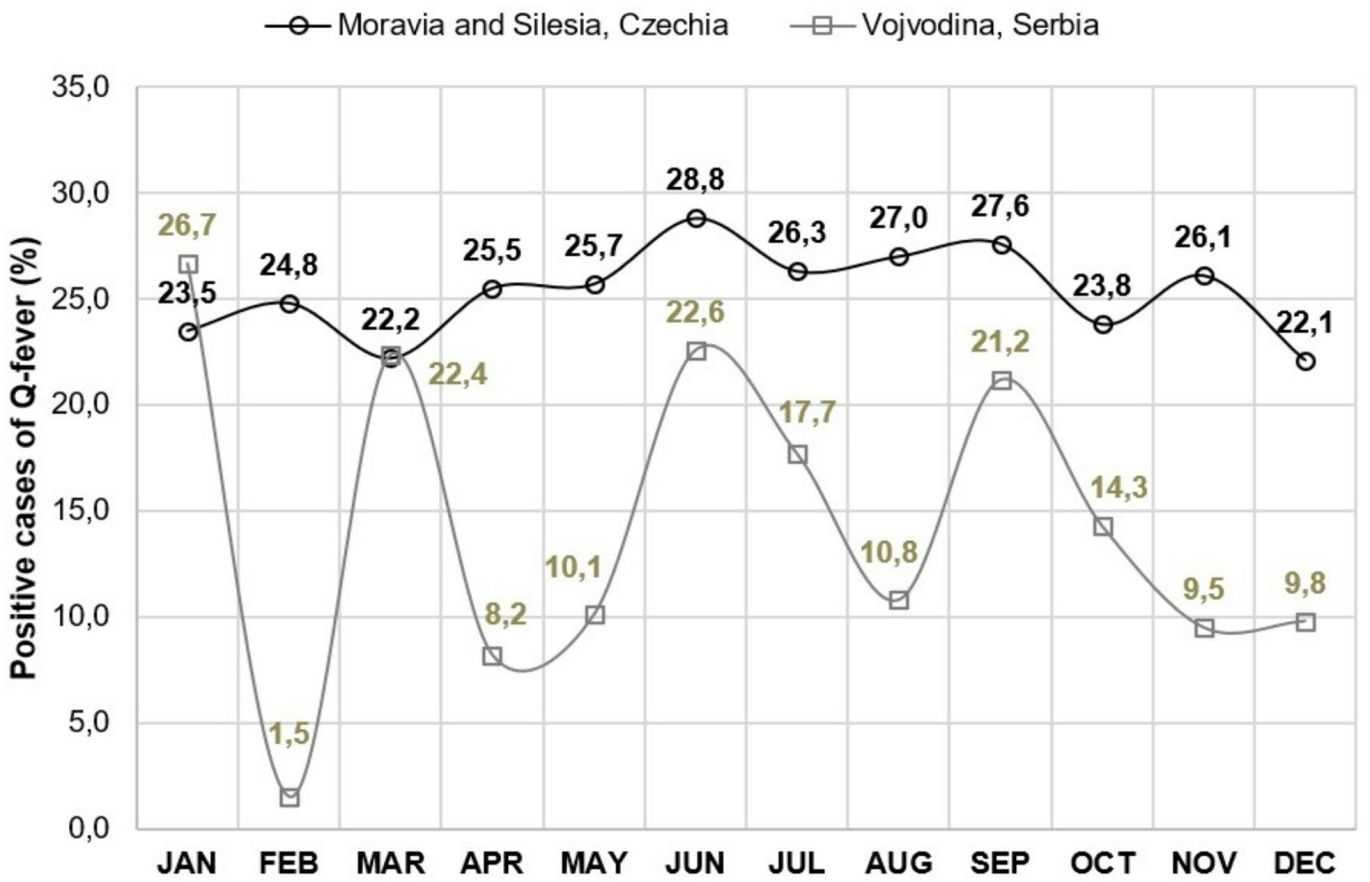
Comparison of mean monthly proportion of *Coxiella burnetii* seropositivity (%) in Moravia/Silesia (Czech Republic) and South Bačka and Srem districts of Vojvodina (Serbia), 2011–2018. *JAN - January*,* FEB - February*,* MAR - March*,* APR - April*,* MAY - May*,* JUN - June*,* JUL - July*,* AUG - August*,* OCT - October*,* NOV - November*,* DEC – December*.

While the number of seropositive animals remained stable in the regions of Moravia and Silesia, there were notable fluctuations in the two districts of Vojvodina (Srem and South Bačka). Here, the mean percentage of seropositive cases varied from 1.5 in February to peaks of 21.2 to 26.7, occurring notably in January, March, June, and September (Fig. 2).

### Q fever in human population

Despite the high seroprevalence of the pathogen in cattle, only three of the five confirmed human Q fever cases, reported in the Czech Republic during 2011–2018, occurred in Moravia and Silesia, including one autochthonous case recorded in South Moravia in 2012 (Table 3). The remaining two cases were imported from Slovenia ( 2016) and Greece (i2018) and involved middle-aged males.

**Table 3:** Demographic characteristics of reported human Q fever cases during the 2011–2018 period in Moravia and Silesia regions, Czech Republic.

Region	Sex	Age	First Symptoms (Date)	Diagnosis	Import	Autochthonous	Country of origin
South Moravia	M	10 m	15.05.2012	A78	No	Yes	-
Moravian-Silesian	M	35–39	21.06.2016	A78	Yes	No	Slovenia
Zlín	M	23	24.09.2018	A78	Yes	No	Greece

Table 4 delineates the comprehensive data on human Q fever cases and outbreaks documented in the Srem district, Vojvodina and Serbia from 2011 to 2018, while no cases were recorded in the South Bačka district during this period. Overall, 1224 human sera samples were tested during the study period in Vojvodina. Across this period, the reported cases in Vojvodina annually ranged from 7 to 71, summing to a total of 231 cases with an incidence rate of 1.5/100.000, approximately 2.5-fold higher than the national average (0.6/100.000). Of these,76 (32.9%) cases were registered in Srem .The majority of human Q fever cases in Vojvodina, were laboratory confirmed (97,4%) and only six human cases were classified as probable cases. Concurrently, outbreaks varied from 0 to 2 per year, culminating in 6 outbreaks in total with half of them (3) occurring in the Srem district (one each in 2011, 2012, and 2017). Notably, the percentage of cases reported in outbreaks fluctuated between 26.9% and 67.6% (overall 44.6% in Vojvodina) whereas in the Srem district, the vast majority of cases (92.1%) were registered in outbreaks. Furthermore, the transmission route of *Coxiella burnetii* in outbreaks in the Srem district was predominantly airborne, being identified in one outbreak, while both airborne and direct contact transmission were reported in two outbreaks (2011 and 2017) (Table 4).

**Table 4:** Reported cases of human Q fever during the 2011–2018 period in Srem district, Vojvodina and the whole of Serbia.

Surveillance indicator	Area	2011	2012	2013	2014	2015	2016	2017	2018	Total	Average
Number of cases	Srem	6	45	1	1	1	0	22	0	**76**	9.5
	Vojvodina	8	71	38	17	25	26	39	7	**231**	28.9
	Serbia	8	74	102	18	28	34	39	8	**311**	38.9
Number of outbreaks	Srem	1	1	0	0	0	0	1	0	**3**	0.4
	Vojvodina	1	2	1	0	0	1	1	0	**6**	0.8
	Serbia	1	2	2	0	0	2	1	0	**8**	1
% of cases reported in outbreaks	Srem	83.3	95.6	0	0	0	0	100.0		**92.1**	-
	Vojvodina	62.5	67.6	55.3	0	0	26.9	56.4	0	**44.6**	-
	Serbia	62.5	64.9	83.3	0	0	50.0	56.4	0	**57.9**	-
The presumptive route of *C. burnetii* transmission^1,2^	Airborne	0	1	0	0	0	0	0	0	**1**	NA^3^
	Airborne and direct contact	1	0	0	0	0	1	0	0	2	

^1^Based on the clinical, epidemiological, and laboratory findings.

^2^Refers to outbreaks recorded in Srem district of Vojvodina.

^3^Not applicable.

**Fig. 3:**
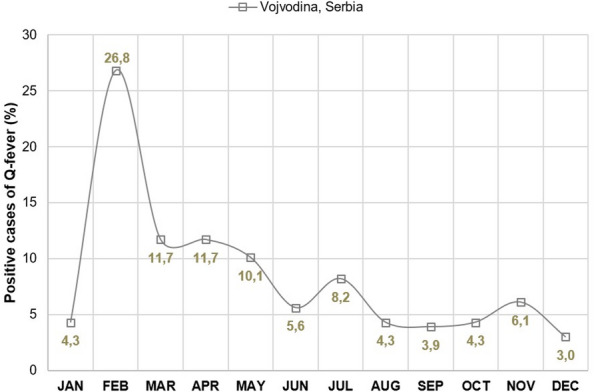
Mean monthly proportion of human Q fever cases (%) in Vojvodina (Serbia), 2011–2018.

Notably, a significant proportion of Q fever cases in Serbia were concentrated within Vojvodina accounting for the majority of cases. Within this province, cases in the Srem district were reported in six of the eight years under study. Additionally, 75% of all recorded outbreaks unfolded within Vojvodina, with outbreaks documented in five of those years. Seasonal analysis unveiled a notable surge in morbidity observed from January through May (64,6% of all cases), reaching its zenith in February (Fig. 3).

In Vojvodina, gender distribution was in favor of men (74%). The overall male-to-female ratio was 2.8:1. The four fifths of all patients belonged to the working-age population (age group 20–59 years). Specifically, the age group 30–39 years suffered the most, accounting for 25,5% of all cases. A similar demographic structure of patients was recorded in the Srem District.

**Fig. 4:**
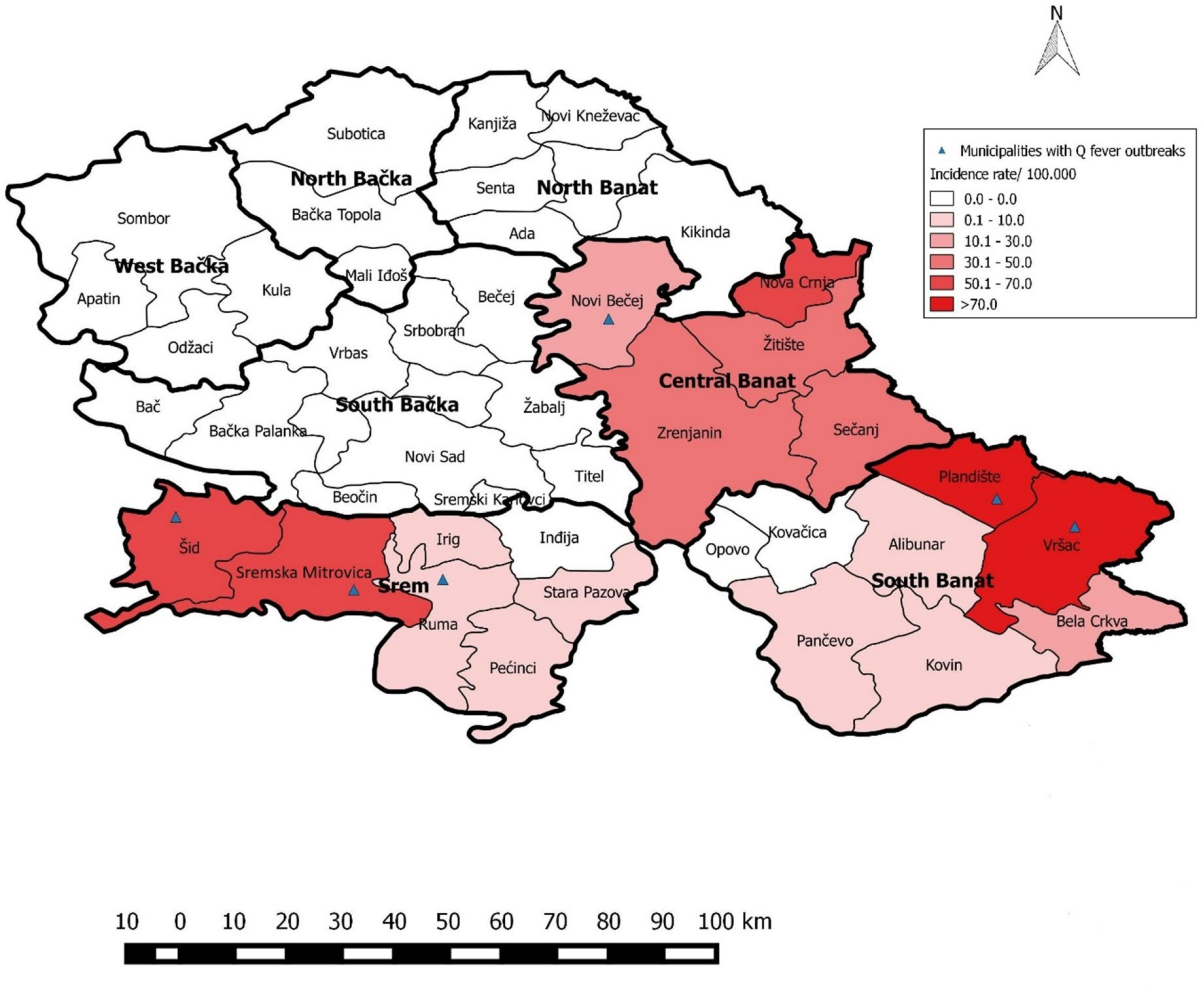
Distribution of incidence rates of Q fever cases per 100,000 population across districts and municipalities, in Vojvodina, Serbia, 2011–2018.

Incidence of human Q fever in Vojvodina was highest in the southwest, near the borders with Croatia and Bosnia and Herzegovina, as well as in the eastern regions bordering Romania (Fig. 4). Notably, the districts of Southern Banat, Srem, and Central Banat exhibited the highest incidence rates within Vojvodina. Over the eight-year study period, the overall incidence of Q fever in Vojvodina was 1.5 per 100,000 population, with the highest rates recorded in Central Banat (5.4/100,000), followed by Southern Banat (3.4/100,000) and Srem (3.1/100,000), while no cases were reported in the remaining districts. Additionally, heightened incidence was identified in the Šid municipality of Srem district, where Q fever was prevalent among goats (Fig. 4).

## Discussion

The maintenance and increase in Q fever seroprevalence among cattle in the Moravia and Silesia, may be related to the growing livestock population^28^, that impacts the living conditions of the animals, leading to overcrowding, thereby facilitating the rapid dissemination of the infectious agent. The use of contaminated manure as fertilizer may cause infection in animals and humans. In addition, silent circulation of *Coxiella burnetii* among wild small mammals^32^, could have contributed to its maintenance in nature.

In 2017, the overall seroprevalence in the EU/EEA was 8.6% in cattle and 9.2% in sheep and goats with large international disparities^33^. Our findings in Vojvodina indicate that sheep and goats may be less seropositive than cattle but so far, they have so far served as the most important source of human *Coxiella burnetii* infection, particularly in an endemic areas, such as Srem District^34,35^. Unlike industrial cattle farms in Czech Republic, that benefit from veterinary oversight and regular reporting, preventive measures in Serbia are commonly integrated into traditional family-owned household settings^36^, where seropositive animals may go unnoticed and may serve as a main reservoir for infection in humans^35,37^.After the civil war in former Yugoslavia (1991–1995), a serious decline in livestock was observed, followed by the termination of nomadic herding and a significant decline in the incidence of Q fever (< 5 per 100,000 inhabitants) in Vojvodina^15,20^. Like the Czech Republic, in Vojvodina, nowadays seropositive cattle are discovered precisely on farms with large traffic of animals to slaughterhouses, despite high levels of bio-safety measures and supervision by veterinarians^34,35^. The different animal sampling strategies in the Czech Republic and Serbia may have influenced the observed *Coxiella burnetii* seroprevalence. In the Czech Republic, targeted sampling of animals shortly after abortion likely detects a higher proportion of seropositive animals, whereas in Serbia, inclusion of asymptomatic animals during outbreak investigations and annual health monitoring provides a broader and potentially lower estimate of herd-level seroprevalence, consistent with previous studies showing that sampling design strongly affects apparent prevalence in livestock populations^38^.

Unlike the consistent seroprevalence of animal Q fever in Moravia and Silesia, distinct peaks were observed in January, March, June, and September in Vojvodina. This variation can be attributed to irregular veterinary examinations and the absence of permanent registration systems for seropositive animals on small farms^39^. The peak incidence of Q fever in humans in Vojvodina is in February, which correlates with the lambing season^15,35^. Moreover, the majority of human Q fever cases in the Srem district of Vojvodina were registered from January to May, which is associated with the kidding and lambing of goats and sheep in these months, and increased frequency of human exposure to potentially seropositive animals^15,35^. Airborne transmission by inhalation of contaminated dust and aerosols was the most likely route of transmission of *Coxiella burnetii* among the majority of patients in the Q fever outbreak in Noćaj, Srem, in January 2012, when unusually dry and windy weather conditions favored the spread of the pathogen^40^. In the Q fever outbreak in 2017, originating from a goat farm in Srem, most patients were infected during direct contact with animals, and a small proportion by the air-borne route^35^.

Surveillance of human Q fever in the EU/EEA is predominantly passive and case-based^25,33^. The EU/EEA notification rate of confirmed Q fever cases as of 0.2/100 000 population, has been reported between 2015 and 2019^19^. In the period 2014–2018, the average incidence of Q fever in humans in Serbia was twice as high as in the EU (0.4/100,000, range 0.1 and 0.6/100,000) and was highest in Vojvodina (average 1.2/100.000, range 0.9-2.0/100,000 population)^20,21^. Despite higher seroprevalence among animals in Moravia and Silesia, human cases have been exceptionally rare. This phenomenon may be attributed to minimized human contact with farm animals in the Czech Republic, where large industrial farms predominate, averaging 24 hectares in size^41^. The utilization of farm automation and skilled labour may further mitigate the risk of zoonotic infections^42^. Additionally, the strategic location of large farms away from populated areas, coupled with the typically humid and high-rainfall weather conditions during winter calving in seasonal herds, may reduce the likelihood of airborne transmission^43,44^. However, on dairy farms with year-round calving, this seasonal effect is less pronounced, and other environmental and management factors influence the risk of transmission. Furthermore, animals in the Czech Republic have not been vaccinated against Q fever on a large scale, even though the vaccine was available^43^.

Meanwhile, in Vojvodina, human Q fever outbreaks are notably frequent^20,40^. Despite the detection of seropositive animals in the South Bačka, no human cases of Q fever were reported during the study period. In contrast, one-third of human cases and half of the outbreaks (3 out of 6) in Vojvodina occurred in the Srem, indicating that this area represents an endemic focus for the disease. The high incidence of human Q fever in Srem may be related to local small ruminant farming practices, which increase the likelihood of environmental contamination and human exposure to *Coxiella burnetii*, particularly during parturition. This is consistent with observations that infection with Coxiella burnetii in the Srem is predominantly present in sheep populations, particularly in the western part of the district and in areas surrounding mountain Fruška Gora, where flocks are often kept on pasture year-round and may come into direct or indirect contact with other animals, facilitating the spread of infection. By comparison, the absence of human cases in South Bačka is likely explained by the predominance of seropositive cattle, which are generally considered less important reservoirs for human infection than seropositive sheep and goats, especially in settings where small ruminants are the primary source of environmental contamination. Additionally, the introduction of infected animals into densely populated settlements may represent an important risk factor for the occurrence of human outbreaks. This trend aligns with the significant presence of goats and sheep, which serve as primary reservoirs, in Serbia, compared to the Czech Republic. As of December 1, 2017, in Serbia the total livestock count stood at 898,650 cattle, 1,704,192 sheep, and 182,558 goats^45,46^. Despite existing recommendations, farmers often work without external veterinary assistance and directly come into contact with newborn lambs and placentas of potentially seropositive sheep and goats without sufficient protective equipment, leading to Q fever infection^35,47^.

The gender distribution favoring men, can be elucidated by the fact that men often undertake more strenuous agricultural tasks, such as slaughtering and cleaning livestock pens^48^. Besides, the male predominance may be due to the hypothesized protective role of estradiol on the immune response to infection caused by *Coxiella burnetii*, which is why women often remain asymptomatic^49^. The transmission of the pathogen may be linked to the movement of animals across borders, as outlined by Debeljak et al.^50^. Additionally, under-reporting of abortions in domestic animals was also observed, which is why there is occasional late response in case of disease outbreaks in humans^35,47^.

A reduced prevalence of *Coxiella burnetii* in the uterine fluid, vaginal mucus and milk of vaccinated dairy goats and sheep, contributed to more efficient Q fever outbreak control in the Netherlands (2008–2009)^51^. However, the widespread use of livestock vaccination has not taken off because the effectiveness of vaccination against biosecurity risks has not been evaluated^52^. Mandatory vaccination of animals against Q fever may be considered in regions with unfavourable climatic conditions beyond human control. For example, in Serbia, vaccinating livestock could be a potential strategy. Furthermore, it would be prudent to introduce international recommendations to prevent the transmission of infectious agents from neighbouring regions^53^.

### Authors’ remarks from one health perspective: the case of EWARS for Q fever outbreaks

Climate change has altered the frequency and intensity of meteorological events, subsequently leading to a shift in the climate-sensitive zoonotic disease transmission dynamics including the Q fever^54,55^. The emergence and re-emergence of Q fever outbreaks are influenced by the interaction between changing climate, animal and human systems. These are fast-spreading diseases with epidemics that overburden already stretched health systems, threatening the stability of societies, and becoming leading causes of morbidity in human and animals in Europe and elsewhere. The implications for future transmission of these diseases are both extremely important and highly uncertain due to data limitations and methodological challenges when integrating climate-driven disease models and climate change projections. Climatic factors such as temperature, precipitation, outdoor dust level and wind speed, along with socio-demographic factors such as population migration, urbanization, poor sanitation and hygiene, and poor healthcare services favor transmission of such diseases^56^.

In both the Czech Republic and Serbia, existing serotype surveillance are fragmented and siloed across various sectors and disease testing is often neither complete in disease scale and scope nor capturing other key climate and environmental intelligence. This important gap leads to delayed risk recognition, primarily reactive outbreak responses and missed opportunities for anticipatory actions that could save lives and resources. Recognizing this critical need of moving from the mere disease detection to case prediction in ‘time’ and ‘space’, this paper proposes a crucial early warning and response system (EWARS) application to prevent the spread to human. Other reports from EU suggested the use of PCR materials together with laboratory-based indicators such as the vaginal swabs as early warning predictors, however their review had concluded huge financial and time cost associated with this data collection^57^. In our paper, we direct the attention to the use of meteorological and if available serotype data as prime predictors instead, which are free and timely accessible mainly through open-access satellite meteorological data^58^. To further ensure effective functions, EWARS should be perceived as an information system designed to support decision making of national and local-level institutions and especially to enable vulnerable groups in the society to take actions to mitigate the impacts of an impending risk. With this in view, EWARS should be a time- and space-function for informing of probable disease outbreaks, but also integrated within existing surveillance and control systems to improve coordination among relevant stakeholders including the national and local animal management agencies that assess risk and develop response strategies, and the public communication channels used to disseminate warning information^59^.

### Limitations

In discussing the findings of our research, it’s essential to acknowledge some limitations. Firstly, the observational design of our study allowed us only to generate hypotheses about the possible causes of Q fever in human population along with the occurrence of seropositive animals but could not confirm causality. In addition, other animals, possible reservoirs of *Coxiella burnetii*, such as small mammals and ticks, were not included in the study, nor were environmental samples collected. Secondly, the data used in our analysis relied heavily on official surveillance data and previous research findings, that may also be subject to limitations such as underreporting, misclassification, or incomplete data collection, thus affecting the accuracy and generalizability of our results. The diagnosis of Q fever is limited by the availability of laboratory testing and reporting, so it could be that most of asymptomatic infections are missed, leading to an underestimation of the official reported incidence rates of Q fever in both humans and animals. Thirdly, a key limitation of this study is that animal data were available for only two of the seven districts in Vojvodina (Srem and South Bačka), which limits the ability to generalize findings on the epizootic situation across the entire province. Other variables besides examined livestock and demographics, such as human behavior, healthcare infrastructure, and socioeconomic conditions may also influence disease dynamics. Moreover, our study focused on specific regions, which may limit the generalizability of our findings to other geographical areas or national level. Finally, while we have proposed potential explanations for the observed trends in Q fever incidence, further empirical investigations are needed to validate these hypotheses.

## Conclusion

Despite high seroprevalence of *Coxiella burnetii* in cattle, in Moravia and Silesia, there were no indigenous human cases of Q fever, which is most probably a consequence of less human exposure due to predominance of large industrial farms located far from populated areas. Moreover, the role of wet weather and high rainfall during the calving season in winter, in reducing the likelihood of airborne dust transmission in Czech study area can be further studied. On the contrary, our results show that Vojvodina is still an endemic area for Q fever but with large regional disparities. Sources of infection are primarily small ruminants, despite higher seropositivity in cattle. Systematic and continuous serological monitoring of animals would allow a better understanding of the distribution and more effective control of Q fever in the region. Given that the study focused on specific regions, it can serve as a cornerstone for a future comparative study that would use national surveillance data from Serbia and one or more countries with more effective control strategies. Considering the importance of the environment in the epidemiology of Q fever, a future national study should also analyze samples taken from the environment. This study underscores the importance of adopting a One Health perspective to effectively understand and mitigate the impact of zoonotic diseases like Q fever. By integrating insights from epidemiology, environmental science, and veterinary medicine, comprehensive strategies to prevent and control disease transmission may be effective in safeguarding the health and well-being of both human and animal populations.

## Data Availability

Data is provided within the manuscript.

## Abbreviations

ECDC: European Center for Disease Prevention and Control
IPHV: Institute of Public Health of Vojvodina
IPHS: Institute of Public Health of Serbia
ELISA: Enzyme-linked immunosorbent assay
CFT: Complement fixation reaction
PCR: Polymerase chain reaction
EEA: European economic area
EU: European union
